# Crosstalk of the Insulin-Like Growth Factor Receptor with the Wnt Signaling Pathway in Breast Cancer

**DOI:** 10.3389/fendo.2015.00092

**Published:** 2015-06-09

**Authors:** Lauren M. Rota, Teresa L. Wood

**Affiliations:** ^1^Department of Pharmacology, Physiology and Neuroscience, New Jersey Medical School and Cancer Center, Rutgers University, Newark, NJ, USA

**Keywords:** IGF, Wnt, beta-catenin, insulin receptor, mouse models, IGF-1R, IGF-II, TNBC

## Abstract

The insulin-like growth factor system has long been considered a pathway that promotes cell proliferation, survival, and transformation, and is thus a promoter of tumorigenesis. However, recent failure of clinical trials for IGF-1R inhibitors reveals the need for a better understanding of how this pathway functions in specific tumor subtypes. Ongoing studies are designed to uncover biomarkers and downstream targets to enhance therapeutic strategies. Other approaches in specific tumor models reveal complex interactions between IGF signaling and other tumor initiating pathways. Here, we review relevant background and recent studies suggesting that inhibiting the IGF-1R can amplify Wnt and Notch signaling pathways in a model of triple negative breast cancer.

## IGF System in Breast Cancer

The insulin/IGF system has very potent mitogenic, survival, and pro-migratory properties for breast cancer cells *in vitro* and in animal models [for reviews, see Ref. (1–3)]. Moreover, high serum levels of IGF-I are correlated with an increased risk for breast cancer, particularly in premenopausal women (4, 5). Collectively, these findings led to development of multiple monoclonal antibodies designed to target the IGF-1R or the IGF ligands, and tyrosine kinase inhibitors to target both the IGF-1R and insulin receptor (IR) [for reviews, see Ref. (6, 7)]. Despite the preclinical findings, the usefulness of disrupting IGF-1R/IR signaling in clinical trials has been less than promising and in some cases has led to worse outcomes (6, 7).

The failure of these trials illuminates the need to better understand patient cohorts that will best be served by disrupting the IGF signaling pathway. Recent studies have demonstrated that an “IGF gene signature” correlating to a set of genes that are up and down regulated by IGF-I is present in human breast cancers, specifically luminal B and triple negative breast cancer (TNBC) (8). Another approach to establish the function of IGF-1R in different types of breast cancers is to disrupt IGF-1R in mouse tumor models with distinct phenotypes. Early studies reported that expression of the IGF-1R predicted a favorable phenotype and a correlation with estrogen receptor (ER) expression (9, 10). Numerous studies have further confirmed crosstalk between the ER and IGF-1R [for reviews, see Ref. (11, 12)]. Consistent with these data, loss of IGF-1R has been associated with breast tumor progression into a more undifferentiated phenotype (13). The studies establishing the IGF-1R as growth promoting for breast cancers suggests some complexity concerning IGF-1R function in breast cancers. One question that has not been well addressed is whether IGF-1R has distinct functions in breast tumors depending on other active signaling pathways and/or the specific mutation(s) or oncogene driving the tumor. We have recently begun to examine this question using a mouse model of TNBC, the *MMTV-Wnt1* mouse.

## The Wnt Signaling Pathway in Mammary Tumorigenesis in Mice

A variety of studies support the conclusion that Wnt pathway hyperactivation contributes to mammary/breast cancers in rodents and humans [for reviews, see Ref. (14–16)]. The Wnt pathway in mammals was first investigated by Nusse and Varmus in 1982, with the observation that overexpression of Wnt-1 in the mammary gland from the mouse mammary tumor virus (MMTV) promoter resulted in mammary hyperplasias by early puberty and mammary tumors between 3 and 8 months (17, 18). The *MMTV-Wnt1* tumor model has been well-characterized as a basal tumor model (19–22). Using flow cytometry markers to label the mammary epithelial lineages in preneoplastic epithelium, Shackelton and colleagues observed a significant increase in the mammary stem cell (MaSC)/myoepithelial (CD24^+^CD29^hi^) population in *MMTV-Wnt-1* epithelium compared to wild type epithelium (23). Other studies suggested that the overexpression of Wnt-1 via the MMTV promoter led to the expansion of mammary progenitor populations, based on the increase in the side population and Sca1+ population (20, 21). The overexpression of Wnt-1 in mammary progenitor cells appears to confer radioresistance (24). More recently, isolation of luminal progenitors from *MMTV-Wnt-1* mice were found capable of reconstituting a mammary gland upon transplantation into a cleared fat pad (25). These studies led investigators to raise the interesting possibility that Wnt-1 regulates the MaSCs, which then change their cell surface phenotype during or after oncogenic transformation (25). However, other studies now support the hypothesis that it is the expanded luminal progenitor population that gives rise to Wnt1 tumors in this mouse model (26). Similarly, recent studies have defined the luminal progenitor as the cell of origin for BRCA tumors in both mouse and human (27–29). These studies strongly support the hypothesis that the basal phenotype of both the Wnt1-driven and the BRCA mutated tumors is acquired subsequent to transformation of a luminal progenitor cell.

## Wnt Signaling in Breast Carcinogenesis

**Figure 1:**
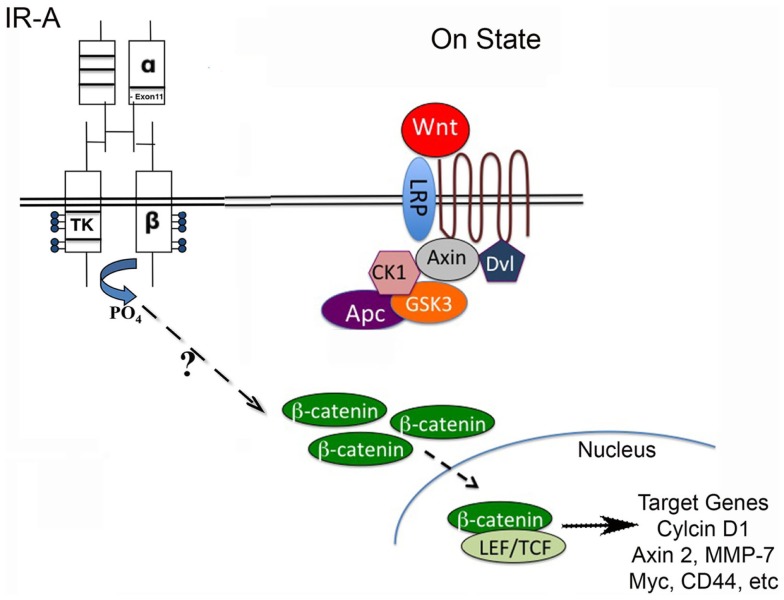
**Wnt/β-catenin pathway regulates stem cell pluripotency and cell fate decisions during development**. The Wnt pathway is activated when Wnt ligand binds to a Frizzled receptor, which then is brought into complex with the co-receptor LRP5/6. The activation of the Wnt pathway leads to stabilization of β-catenin through inactivation of the destruction complex (containing Axin, CK1, Gsk3β, and Apc). β-catenin can then translocate to the nucleus and interact with LEF/TCF to regulate Wnt target genes. The stimulation of the insulin receptor isoform A (IR-A) in IGF-1R null (R-/IR-A) fibroblasts by insulin was shown to increase levels of β-catenin through a mechanism that is currently unknown.

In breast cancer, Wnt signaling is activated in the absence of downstream mutations and can occur via autocrine or paracrine mechanisms (30, 31). A multitude of Wnt ligands and Fzd receptors are overexpressed in breast cancer cell lines and primary human breast tumors (32–34). Secreted frizzled-related protein 1, a negative regulator of the Wnt pathway, is lost in 46–80% of breast cancers and is associated with a poor prognosis (35). Up to 50% of breast tumors have hypermethylation of the adenomatous polyposis coli (*APC*) promoter, and transcript loss leads to hyperactivation of the Wnt pathway (36). *APC* is a negative regulator of β-catenin (see Figure 1). Thus, hyperactivation of the Wnt pathway is common in breast carcinomas. More recently, TNBCs have been classified into six subtypes based on gene expression profiles; Wnt pathway activation was identified in several of the TNBC subtypes (37).

The Wnt signaling pathway is complex and there are still many questions about how to interfere with this pathway in breast and other cancers. Several therapeutic treatments are being explored, such as monoclonal antibodies that target Wnt ligand–receptor interaction and small molecule inhibitors designed to down-regulate Wnt secretion; for review, see Ref. (38, 39). Understanding pathways that interact with and potentially inactivate Wnt signaling, however, is of vital importance to develop more effective therapeutic treatments.

## IGF-1R and Wnt Pathway Crosstalk

In recent studies, we attenuated IGF-1R signaling in the *MMTV-Wnt1* tumor model and demonstrated decreased tumor latency, increased tumor incidence, and development of a metastatic tumor phenotype (40). These results were surprising given that the IGF-1R has been considered predominantly a positive-mediator of breast cancer growth. Moreover, transgenic mice overexpressing or with constitutive activation of the IGF-1R develop mammary tumors as early as 8 weeks of age (41–43). A recent study further demonstrated that the ability of a Wnt pathway inhibitor to reduce tumor growth in the MMTV-Wnt1 model was due to upregulation of the IGF-binding protein-5 (44). The authors of this study further suggested that part of the mechanism for IGFBP-5 in reducing tumor volume after acute treatment with the Wnt pathway inhibitor was due to down-regulation of IGF-1R signaling. However, our data suggest an opposing role for the IGF-1R in the context of active Wnt signaling in a chronic model and support the conclusion that the IGF-1R is protective against Wnt-mediated oncogenesis. In the remainder of this review, we focus on the possible mechanisms for and implications of our data on the function of the IGF-1R in the *MMTV-Wnt1* tumor model and in TNBCs.

One explanation for our findings is that the loss of the IGF-1R alters mammary epithelial lineages leading to amplification of the luminal progenitor population responsible for Wnt-mediated tumors. Support for the IGF-1R in maintaining normal MEC lineage distribution is our observation that the attenuation of the IGF-1R in the normal mammary epithelium leads to an accumulation of luminal progenitors that are enriched in the CD61^+^CD29^lo^ flow cytometry profile (40). The mechanism for this shift in lineage is likely due to the dysregulation of Notch signaling with down-regulation of the IGF-1R in both normal mammary epithelium and the *MMTV-Wnt1* hyperplasias (40). In addition, we showed a decrease in Elf-5 expression in bigenic tumors compared to *MMTV-Wnt1* tumors. Loss of *Elf-5* leads to an increase in the CD61+ luminal progenitors and hyperactive Notch signaling (45, 46). Finally, previous data demonstrated that Wnt-mediated oncogenic conversion of breast epithelial cells is Notch-dependent (33). Taken together, these data support the hypothesis that dysregulation of Notch signaling with down-regulation of IGF-1R signaling leads to accelerated tumor development in the presence of active Wnt signaling. How the attenuation of IGF-1R signaling leads to dysregulation in Notch signaling is not entirely clear. Previous studies have demonstrated that the IGF-1R is a substrate of, and upregulated by, Notch1 signaling in T-ALL cells (47) and in lung adenocarcinomas (48). However, no studies to date have shown regulation of Notch signaling by IGF-1R activation.

## IR-A in Wnt-Mediated Tumorigenesis

A second potential mechanism for enhanced tumorigenesis of *MMTV-Wnt1* tumors with decreased IGF-1R signaling is through enhanced IR signaling, in particular through the IR-A isoform [for review of IGF-1R/IR isoform signaling, see Ref. (49, 50)] (see Figure 1). Prior studies from the Yee laboratory demonstrated that down-regulation of IGF-1R signaling in a variety of breast cancer cell lines increases insulin sensitivity by increasing cell surface expression of holo-IR due to a reduction in hybrid IR/IGF-1R cell surface expression (51). Previous studies have shown that the overexpression of IR-A is increased in breast tumor samples (52). Furthermore, IR-A has been suspected as one of the possible mechanisms responsible for resistance to IGF-1R targeted therapies (53, 54).

Consistent with these findings, we found that the *MMTV-Wnt1* tumors with decreased IGF-1R signaling have an increased IR-A:IR-B ratio, as well as increased expression of IGF-II, a high-affinity ligand for the IR-A. To determine if IGF-II signaling through the IR-A can activate canonical Wnt signaling, we stimulated IGF-1R null/IR-A overexpressing fibroblasts with IGF-II and analyzed protein levels of β-catenin, that is stabilized by canonical Wnt signaling (Figure 1). We found that levels of β-catenin are increased in a dose-dependent manner by IGF-II stimulation of IR-A expressing fibroblasts (40). The Wnt/β-catenin as well as IR-A signaling pathways have been implicated in stem cell and cancer stem cell renewal [for reviews, see Ref. (31, 55, 56)]. Although both pathways are expressed in similar tissues and seem to have overlapping actions, the interaction between these two pathways remains unclear. Our data suggest a novel role for IR-A in Wnt-mediated oncogenesis in the absence of IGF-1R signaling, and further support the hypothesis that inhibiting the IGF-1R may amplify this signaling cascade.

## Relevance to Breast Cancer

The Wnt pathway is commonly altered in basal-like or TNBC subtypes (37, 57). Our data suggest a complex interaction between IGF-1R/IR and Wnt pathway signaling and support the need for screening breast cancer patients for expression of Wnt pathway components in combination with IGF-1R as well as the IR-A:IR-B ratio and potentially IGF-II to better understand how inhibitors of these pathways might be best employed in different tumor subtypes. It is possible that inhibiting the IGF-1R or downstream signaling will lead to amplification of more undifferentiated tumors containing increased stem/progenitor populations with higher self-renewal potential through induction of Wnt and/or Notch pathway signaling. Dual inhibition of the IGF-1R/IR receptors or downstream signaling targets in the IR-A pathway along with Wnt pathway inhibitors may be beneficial in specific TNBCs. Understanding the mechanism(s) responsible for IR-A stimulation of the canonical Wnt pathway could prove helpful in designing therapeutic targets in subclasses of TNBCs.

## Conflict of Interest Statement

The authors declare that the research was conducted in the absence of any commercial or financial relationships that could be construed as a potential conflict of interest.
